# Supplementary material for: Neutrophil Immunomodulatory Activity of (−)-Borneol, a Major Component of Essential Oils Extracted from Grindelia squarrosa
Source: Molecules. 2022 Jul 31;27(15):4897. doi: 10.3390/molecules27154897 (PMC9369983; doi:10.3390/molecules27154897)
Supplement: Supplementary file 1 [file molecules-27-04897-s001.zip › molecules-1838118-supplementary.pdf]

Table S1. Chiral Separation Parameters for Enantiomeric Compounds from *Grindelia squarrosa* Essential Oils

Compound	α-Pinene	β-Pinene	Borneol	Camphor	Limonene
**Column**	Lipodex G	Lipodex G	Rt-βDEXse	Rt-βDEXse	Rt-βDEXse
**Temp. program**	50 min@35°C; 40°C/min to 180°C; 11.375 min@180°C; total 65 min		0 min@80°C; 1°C/min to 170°C; total 90 min	0 min@40°C; 2°C/min to 200°C; total 80 min	
**Flow**	Helium, 5 mL/min (Average velocity: 77.991 cm/sec)	Helium, 5 mL/min (Average velocity: 77.991 cm/sec)	Helium, 6 mL/min (Average velocity: 75.938 cm/sec)	Helium, 5 mL/min (Average velocity: 63.943 cm/sec)	

Figure S1. α-Pinene Enantiomers from *Grindelia squarrosa* Essential Oils

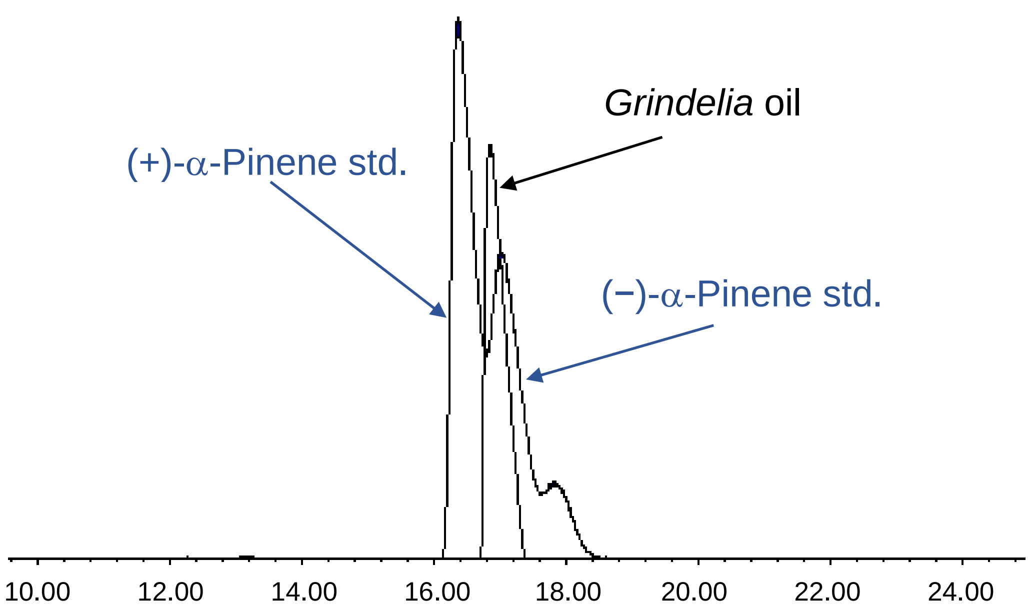

Enantiomer ratio

(+/-)-α-Pinene: (0/100)

Enantiomeric excess (ee): 100% (−)-α-Pinene

Analysis conditions

Column: Lipodex G (*6-Methyl-2,3-pentyl-γ-cyclodextrin added into 60% in polysiloxane*); (25 m, 0.25 mm ID, 0.125 μm film thickness)

Temp. program: 50 min@35°C; 40°C/min to 180°C; 11.375 min@180°C; Total 65 min

Flow: Helium, 5 mL/min (Average velocity: 77.991 cm/sec)

Injection volume: 1 μL (10% in hexane)

Split ratio: 10:1

Injection port Temp.: 250°C

Detector Temp.: 250°C

Figure S2. β-Pinene Enantiomers from *Grindelia squarrosa* Flower Essential Oils

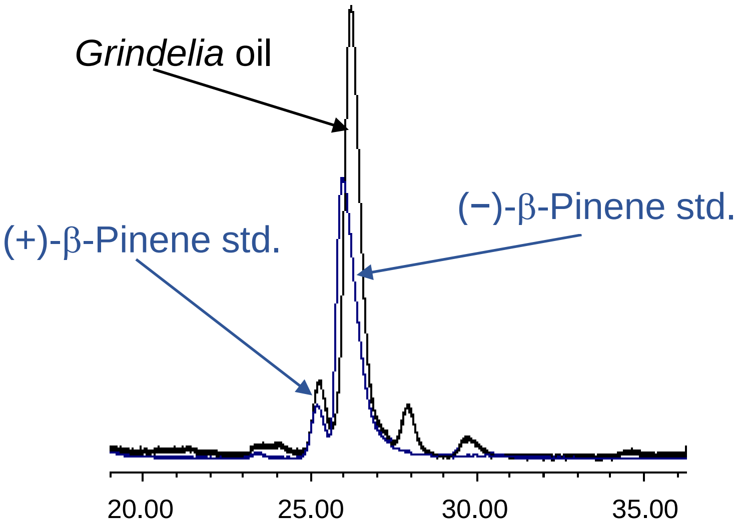

Enantiomer ratio

(+/-)-β-Pinene: (9/91)

Enantiomeric excess (ee): 82% (−)-β-Pinene

Analysis conditions

Column: Lipodex G (*6-Methyl-2,3-pentyl-γ-cyclodextrin added into 60% in polysiloxane*);
(25 m, 0.25 mm ID, 0.125 μm film thickness)

Temp. program: 50 min@35°C; 40°C/min to 180°C; 11.375 min@180°C; Total 65 min

Flow: Helium, 5 mL/min (Average velocity: 77.991 cm/sec)

Injection volume: 1 μL (10% in hexane)

Split ratio: 10:1

Injection port Temp.: 250°C

Detector Tem

Figure S3. Camphor Enantiomers of the *Grindelia squarrosa* Leaf Essential Oils

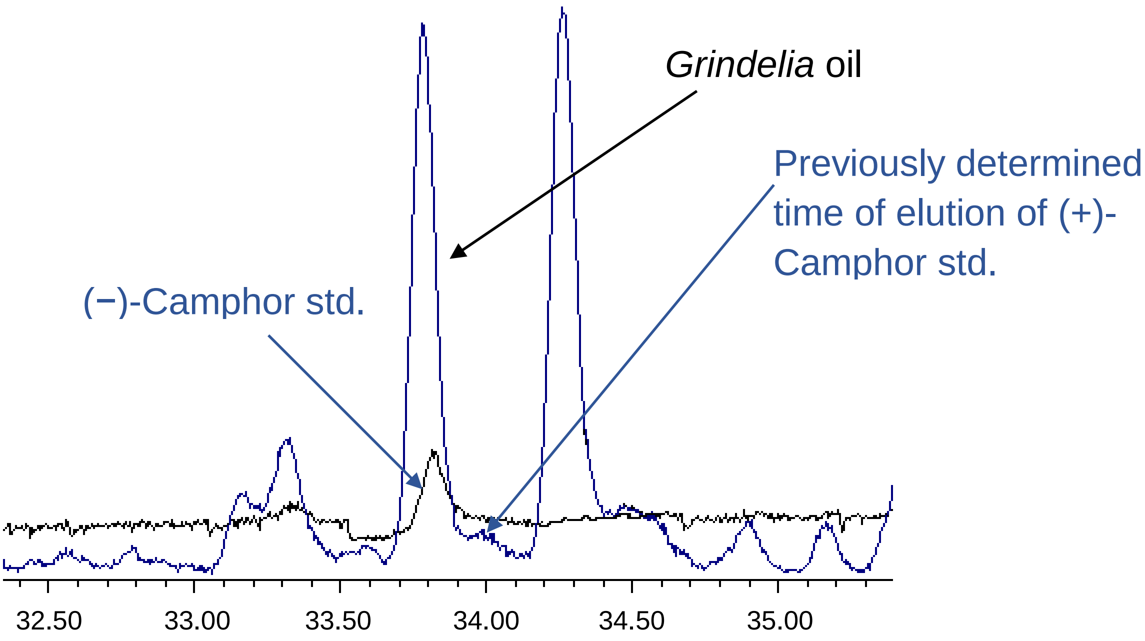

Enantiomer ratio

(+/-)-Camphor: (3/97)

Enantiomeric excess (ee): 94% (−)-Camphor

Analysis conditions

Column: Rt-βDEXse (*2,3-di-O-ethyl-6-O-tert-butyl dimethylsilyl β-cyclodextrin added into 14% cyanopropylphenyl/86% dimethyl polysiloxane*);
(30 m, 0.32 mm ID, 0.25 μm film thickness)

Temp. program: 0 min@40°C; 2°C/min to 200°C; Total 80 min

Flow: Helium, 5 mL/min (Average velocity: 63.943 cm/sec)

Injection volume: 1 μL (10% in hexane)

Split ratio: 10:1

Injection port temp.: 250°C

Detector temp.: 250°C

Figure S4. Borneol Enantiomers of the *Grindelia squarrosa* Flower Essential Oils

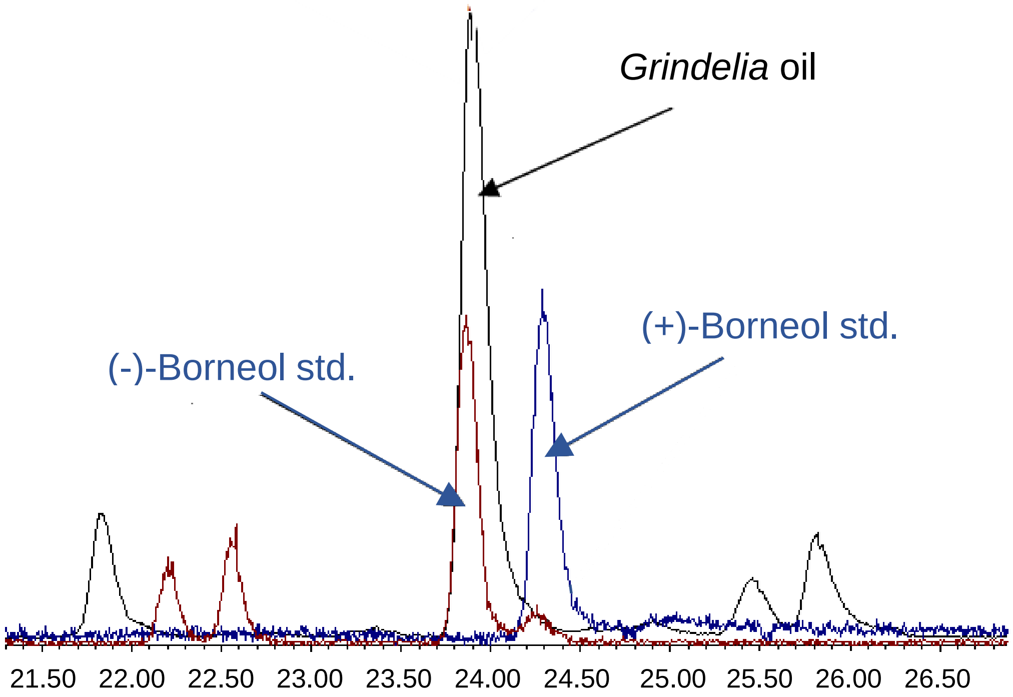

Enantiomer ratio

(+/-)-Borneol: (0/100)

Enantiomeric excess (ee): 100% (−)-Borneol

Analysis conditions

Column: Rt-βDEXse (*2,3-di-O-ethyl-6-O-tert-butyl dimethylsilyl β-cyclodextrin added into 14% cyanopropylphenyl/86% dimethyl polysiloxane*);
(30 m, 0.32 mm ID, 0.25 μm film thickness)

Temp. program: 0 min@80°C; 1°C/min to 170°C; Total 90 min

Flow: Helium, 6 mL/min (Average velocity: 75.938 cm/sec)

Injection volume: 1 μL (10% in hexane)

Split ratio: 10:1

Injection port temp.: 250°C

Detector temp.: 250°C

Figure S5. Limonene Enantiomers of the *Grindelia squarrosa* Essential Oils

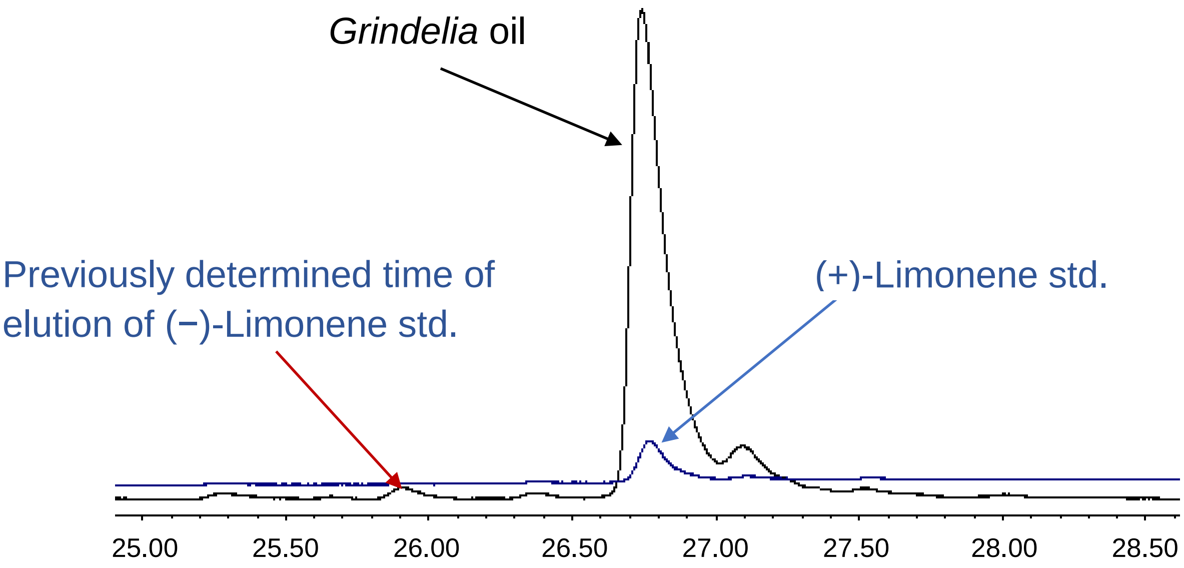

Enantiomer ratio

(+/-)-Limonene: (98/2)

Enantiomeric excess (ee): 96% (+)

Analysis conditions

Column: Rt-βDEXse (*2,3-di-O-ethyl-6-O-tert-butyl dimethylsilyl β-cyclodextrin added into 14% cyanopropylphenyl/86% dimethyl polysiloxane*);
(30 m, 0.32 mm ID, 0.25 μm film thickness)

Temp. program: 0 min@40°C; 2°C/min to 200°C; Total 80 min

Flow: Helium, 5 mL/min (Average velocity: 63.943 cm/sec)

Injection volume: 1 μL (10% in hexane)

Split ratio: 10:1

Injection port Temp.: 250°C

Detector temp.: 250°C
